# Molecular docking analysis and anti-hyperglycaemic activity of Synacinn™ in streptozotocin-induced rats

**DOI:** 10.1039/d0ra04664g

**Published:** 2020-09-18

**Authors:** Nur Syukriah Ab Rahman, Fadzilah Adibah Abdul Majid, Mohd Effendy Abd Wahid, Hassan Fahmi Ismail, Fatahiya Mohamed Tap, Ain Nabihah Zainudin, Siti Nurazwa Zainol, Muzaida Aminah Mohammad

**Affiliations:** Institute of Marine Biotechnology, Universiti Malaysia Terengganu 21030 Malaysia f.adibah@umt.edu.my; Faculty of Chemical Engineering, Universiti Teknologi Mara Bukit Besi 23200 Dungun Terengganu Malaysia; Proliv Life Sciences Sdn Bhd D-1-16, Residensi Bistaria, Jalan Ulu Kelang, Taman Ukay Bistari 68000 Ampang Selangor Malaysia

## Abstract

Synacinn™ is a standardized polyherbal supplement formulated from *Cinnamomum zeylanicum* Blume, *Curcuma zanthorrhiza* Roxb., *Syzygium polyanthum* (Wight) Walp., *Orthosiphon stamineus* Benth. and *Andrographis paniculata* (Burm.f.) Nees. It is designed for the synergistic treatment of diabetes mellitus and its complications. Although the beneficial effects are yet to be verified scientifically, it is traditionally used to improve general health in patients with diabetes. This study aimed to evaluate the anti-hyperglycemic effects of Synacinn™ in a streptozotocin-induced type 1 diabetes rat model. Initially, Synacinn™ was used for *in vivo* acute oral toxicity tests and 14 day repeated dose toxicity tests to determine the toxicity levels. An efficacy study of Synacinn™ was carried out *via* the oral administration of 10, 50, 100, 250, and 250 (b.i.d.) mg kg^−1^ doses to streptozotocin-induced diabetic rats. After 28 days, blood serum was collected to measure the fasting blood glucose, triglyceride, cholesterol, alanine aminotransferase, alkaline phosphatase, creatinine, and uric acid levels. The liver, kidney, and pancreas structures were histopathologically analyzed. *In silico* binding interaction studies of five phytochemicals in Synacinn™ identified *via* HPLC with glucokinase were performed using molecular docking analysis. The results showed that although no mortality was observed during the acute oral toxicity tests, notable damage to the liver and kidney occurred during the 14 day repeated dose testing at Synacinn™ levels of 600 mg kg^−1^ and 2000 mg kg^−1^. Treatment with 250 mg kg^−1^ (b.i.d.) Synacinn™ of the streptozotocin-induced type 1 diabetic rats significantly (*p* < 0.05) improved the fasting blood glucose (59%), triglyceride (58%), cholesterol (47%), alanine aminotransferase (60%), alkaline phosphatase (90%), and creatinine (32%) levels. Synacinn™ also improved the relative weights of liver (35%), kidney (36%), and pancreatic (36%) tissue. Histological analysis showed improvements in the conditions of the central vein of the liver, the kidney Bowman's capsule and glomerulus, and the pancreatic islets of Langerhans. HPLC analysis of a standardized extract identified five active phytochemicals: andrographolide (17.36 mg g^−1^), gallic acid (11.5 mg g^−1^), curcumin (2.75 mg g^−1^), catechin (3.9 mg g^−1^), and rosmarinic acid (5.54 mg g^−1^). Molecular docking studies with glucokinase showed that andrographolide yields the highest binding energy (−12.1 kcal mol^−1^), followed by catechin (−10.2 kcal mol^−1^), rosmarinic acid (−8.6 kcal mol^−1^), curcumin (−7.8 kcal mol^−1^), and gallic acid (−5.6 kcal mol^−1^). These current findings suggest that Synacinn™ at a dose of 250 mg kg^−1^ was non-toxic to rats. A twice-daily 250 mg kg^−1^ dose of Synacinn™ is an effective anti-hyperglycemic agent, lowering blood glucose, triglyceride, and cholesterol levels, and assisting the recovery of organ impairment caused by streptozotocin in type 1 diabetic rats.

## Abstract 2

Synacinn™, a standardized polyherbal supplement, was shown to improve hyperglycemic conditions and related complications in STZ-induced rats.

## Graphical abstract

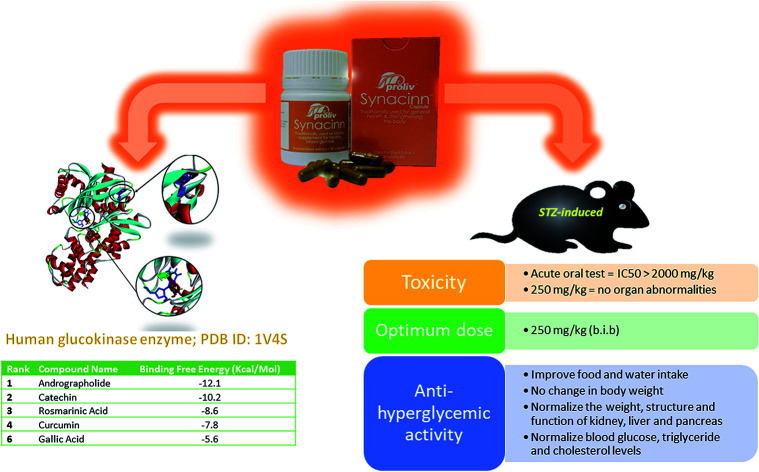

## 1. Introduction

Diabetes mellitus (DM) is becoming the largest global epidemic in the present millennium. National Health and Morbidity Surveys (NHMS) have reported a growing trend in DM prevalence in past decades and, surprisingly, the overall prevalence of DM in Malaysia has more than doubled from 1996 to 2015.^1^ DM can be managed *via* healthy lifestyle practices, such as regular physical activity and a nutritional diet, as well as pharmacotherapy with anti-diabetic drugs. Despite tremendous amounts of research aimed at understanding its pathology, pharmacotherapy still presents a challenge when it comes to managing DM without side effects. The current management of DM, including *via* insulin therapy and various kinds of allopathic drugs, is reported to result in severe side effects and limited efficacy.^2^ Therefore, alternative anti-diabetic drugs need to be researched and developed that have a strong therapeutic impact with minimal side effects.

For diabetes patients, alternative therapies derived from herbal products have attracted much interest. Various herbal products are being developed that provide better glycemic control and are less dependent on insulin injections or synthetic oral drugs. Herbal products are considered as being safer and cheaper and having fewer adverse effects compared with prescribed drugs.^3,4^ For example, *Morus alba* L., *Panax quinquefolius* L. and *Cinnamomum cassia* (L.) J. Presl have anti-hyperglycemic activities and have been approved as anti-diabetic medicines by the Korean FDA.^5^ It is believed that herbal medicines formulated with multiple herbs provide superior effects when compared with similar herbs taken separately. Additionally, DM is favorably managed *via* a combination of herbs (a polyherbal approach) instead of *via* one herb, due to better synergistic effects and fewer side effects.^6^

Synacinn™ is a standardized anti-diabetic polyherbal supplement, and each capsule contains an equal 10 mg concentration of *Cinnamomum zeylanicum* Blume, *Curcuma zanthorrhiza* Roxb., *Syzygium polyanthum* (Wight) Walp., *Orthosiphon stamineus* Benth., and *Andrographis paniculata* (Burm.f.) Nees. This traditional blend is claimed to exert a synergistic effect to remediate DM conditions and associated complications. Multiple data regarding the biological activities of every single herb in Synacinn™ suggest that there is promise for Synacinn™ to be scientifically validated and developed as an alternative agent for the management of DM. Anti-hyperglycemic activities have been reported from *Cinnamomum zeylanicum* Blume,^7–10^*Orthosiphon stamineus* Benth.,^11–14^*Curcuma zanthorrhiza* Roxb.,^15–19^*Syzygium polyanthum* (Wight) Walp,^20–22^ and *Andrographis paniculata* (Burm.f.) Nees.^23–25^ In our preliminary study, Synacinn™ showed low cytotoxicity levels towards the 1.1B4, 3T3-L1, and WRL-68 cell lines.^26^ However, the toxicity and efficacy of Synacinn™ in *in vivo* models are still unknown. The challenge in this current study is to evaluate safety profiles relating to potential toxicity effects *in vivo* and to determine the anti-hyperglycaemic effects of Synacinn™ in reducing the levels of glucose, triglycerides and cholesterol in streptozotocin (STZ)-induced type 1 diabetic rats.

## 2. Results

### 2.1 Toxicity studies

#### 2.1.1 Acute oral toxicity tests

Zero mortality was observed during acute treatment with 2000 mg kg^−1^ Synacinn™. Also, no changes in behavior, breathing, or nervous responses were observed. Fur, eyes, skin, and noses appeared to be normal, and no convulsion, salivation, or diarrhea were observed until day 14. Body weight and relative organ weight (ROW) differences between rats receiving 2000 mg kg^−1^ body weight Synacinn™ and a control group were insignificant (*p* > 0.05).

#### 2.1.2 14 day repeated oral dose toxicity studies

##### 2.1.2.1 Body weight, and food and water consumption

**Fig. 1:**
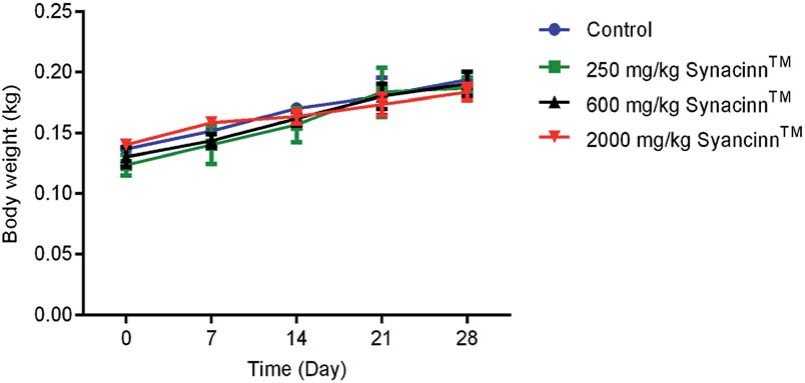
The effect of Synacinn™ on the body weights of rats. Values are mean ± SEM, with *n* = 6 rats per group in the 14 days of repeated dosing and *n* = 3 in the recovery period.

**Fig. 2:**
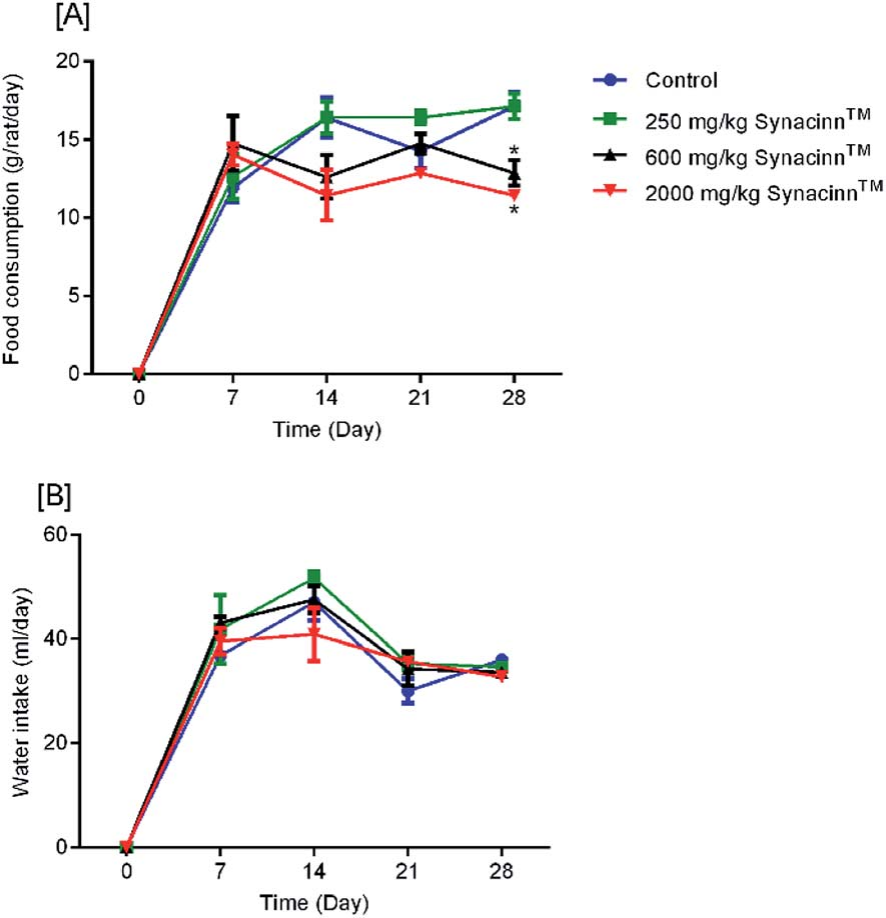
The effects of Synacinn™ on (A) food consumption and (B) water intake. Values are means ± SEM; *n* = 3 rats per group; *: *p* < 0.05 compared to the control group; one-way ANOVA followed by Dunnett's test for *post-hoc* analysis.

In general, no significant changes in body weight (Fig. 1) or food and water consumption (Fig. 2) were observed during Synacinn™ treatment. The body weights of Synacinn™-treated rats increased according to the control rat pattern, with an ∼40% increase from the initial weights. Meanwhile, insignificant differences in food consumption from day 7 to day 21 were measured, with values of around 12 to 16 g per rat per day. However, a significant decrease (*p* < 0.05) in food consumption was observed at day 28 for rats treated with 600 mg kg^−1^ (−25%) and 2000 mg kg^−1^ (−33%) Synacinn™ compared to the control. For water intake, no significant differences were measured compared to the control. However, it was noted that higher water intakes were detected on day 7 and day 14 (40–50 mL per day) compared to day 21 and day 28 (35 mL per day).

##### 2.1.2.2 Relative organ weights and histopathology

**Fig. 3:**
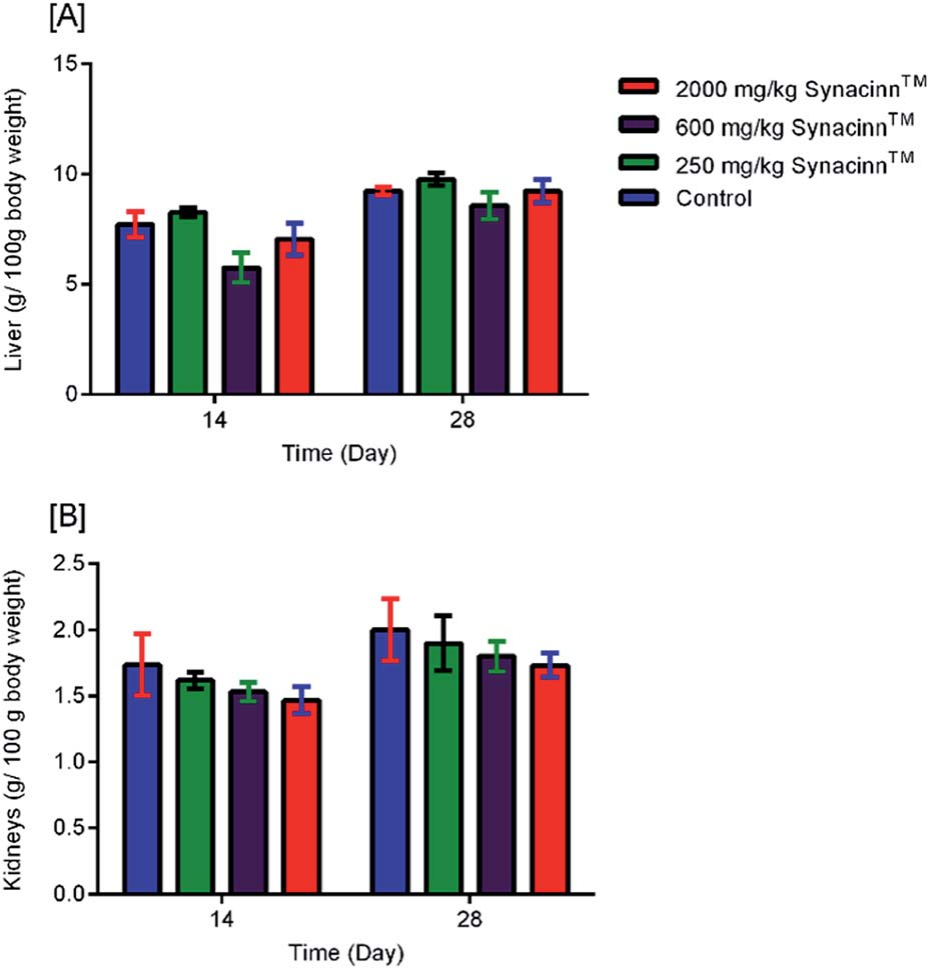
The effects of Synacinn™ on the relative organ weights of rats: (A) relative liver weights; (B) relative kidneys weights. Values are means ± SEM; *n* = 3 rats per group; *: *p* < 0.05 compared to the control group; one-way ANOVA followed by Dunnett's test for *post-hoc* analysis.

**Fig. 4:**
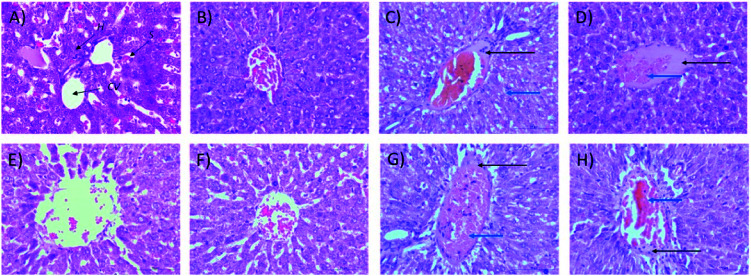
Histopathological analysis of rat liver samples: (A) control after 14 days of repeated doses; (B) 250 mg kg^−1^ Synacinn™ after 14 days of repeated doses; (C) 600 mg kg^−1^ Synacinn™ after 14 days of repeated doses; (D) 2000 mg kg^−1^ Synacinn™ after 14 days of repeated doses; (E) control after a 14 day recovery period; (F) 250 mg kg^−1^ Synacinn™ after a 14 day recovery period; (G) 600 mg kg^−1^ Synacinn™ after a 14 day recovery period; and (H) 2000 mg kg^−1^ Synacinn™ after a 14 day recovery period. CV = central vein; H = hepatocytes; S = sinusoidal spaces.

As shown in Fig. 3, no significant changes (*p* > 0.05) in liver and kidney weights were detected. Histopathological analysis of livers from control and treated rats after 14 day repeated dose studies and a 14 day recovery period is shown in Fig. 4A–D and E–H, respectively. Microscopic examinations of the control rats showed that hepatocytes had a normal arrangement with standard sinusoid and central vein structures (Fig. 4A). Rats treated with 250 mg kg^−1^ Synacinn™ (Fig. 4B) also exhibited normal central vein and hepatocyte structures during this period of study. Meanwhile, Fig. 4C depicts the degenerated hepatocytes (blue arrow) of rats treated with 600 mg kg^−1^ Synacinn™, and the central vein started to show the presence of fibrinous material (black arrow). For rats treated with 2000 mg kg^−1^ Synacinn™ (Fig. 4D), the central vein was filled with fibrinous material (black arrow) and erythrocyte hemolysis was seen (blue arrow). After the recovery period, rats treated with 250 mg kg^−1^ Synacinn™ (Fig. 4F) exhibited a central vein, sinusoids, and hepatocytes with normal appearances. The rats treated with 600 and 2000 mg kg^−1^ Synacinn™ (Fig. 4G and H) showed the presence of fibrinous material (black arrow) on the endothelium wall (blue arrow) and thrombosis.

**Fig. 5:**
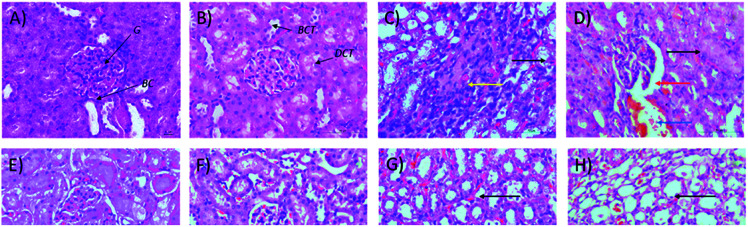
Histopathological analysis of rat kidney samples: (A) control after 14 days of repeated doses; (B) 250 mg kg^−1^ Synacinn™ after 14 days of repeated doses; (C) 600 mg kg^−1^ Synacinn™ after 14 days of repeated doses; (D) 2000 mg kg^−1^ Synacinn™ after 14 days of repeated doses; (E) control after a 14 day recovery period; (F) 250 mg kg^−1^ Synacinn™ after a 14 day recovery period; (G) 600 mg kg^−1^ Synacinn™ after a 14 day recovery period; and (H) 2000 mg kg^−1^ Synacinn™ after a 14 day recovery period. G = glomerulus; BC = Bowman's capsule; PCT = proximal convoluted tubule; DCT = distal convoluted tubule.

Microscopic examinations of kidney samples after 14 days of repeated doses and a recovery period are illustrated in Fig. 5A–H. The control group (Fig. 5A) exhibits a normal glomerulus surrounded by a Bowman's capsule with normal proximal and distal tubules. Similar kidney structures were also detected in rats treated with 250 mg kg^−1^ Synacinn™ (Fig. 5B). Tubular necrosis (black arrow) and hyalinization (yellow arrow) were observed in the rats treated with 600 mg kg^−1^ Synacinn™ (Fig. 5C). In the rats treated with 2000 mg kg^−1^ Synacinn™, the cells lining the tubules experienced hypertrophy (black arrow), with the degeneration of the Bowman's capsule (red arrow) and thrombosis (blue arrow) (Fig. 5D). Meanwhile, after the recovery period, rats treated with 250 mg kg^−1^ Synacinn™ displayed a kidney structure with a normal glomerulus and tubules (Fig. 5F). The rats treated with 600 and 2000 mg kg^−1^ showed a degree of cell necrosis (black arrows) during this period (Fig. 5G and H), indicating the toxic nature of both doses during the recovery period.

### 2.2 Anti-hyperglycemic effects of Synacinn™ in STZ-induced diabetic rats

#### 2.2.1 Food consumption and water intake

**Fig. 6:**
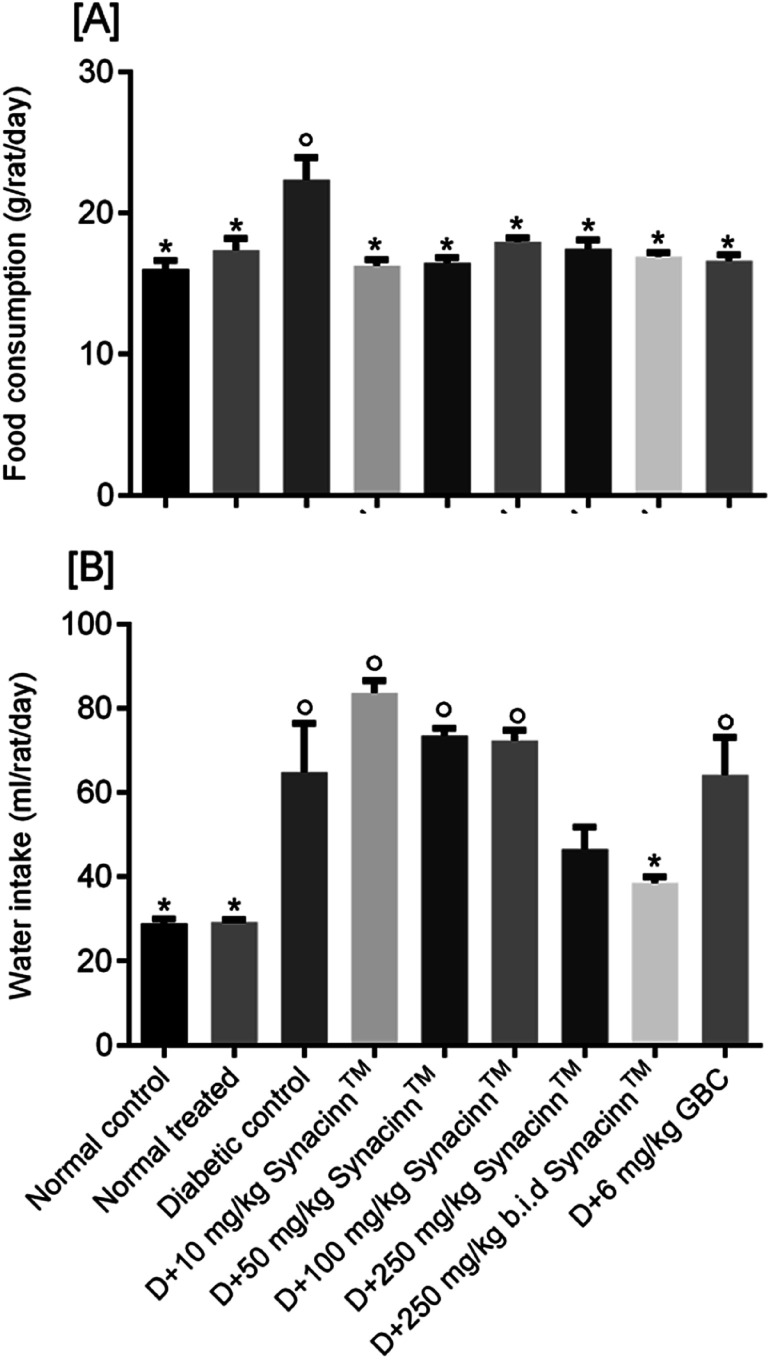
The effects of Synacinn™ on (A) food consumption and (B) water intake by normal and diabetic rats. Values are mean ± SEM; *n* = 6 rats per group; °: *p* < 0.05 compared to the normal control; *: *p* < 0.05 compared with the diabetic control group; one-way ANOVA followed by Tukey's test for *post-hoc* analysis. D = diabetic induced; GBC = glibenclamide.

Polyphagia (excessive hunger) and polydipsia (excessive thirst) are major symptoms of diabetes mellitus.^27^ Referring to Fig. 6A, the administration of STZ significantly increased (*p* < 0.05) food consumption by 40%. Interestingly, the treatment of diabetic rats with 10, 50, 100, 250 and 250 (b.i.d.) mg kg^−1^ Synacinn™ reduced the consumption of food by 38%, 37%, 28%, 31%, and 34%, respectively, back toward normal levels. Treatment with glibenclamide also reduced food consumption by 36%.

For water intake (Fig. 6B), the administration of STZ significantly increased water intake by 124%. Treatment with 250 (b.i.d.) mg kg^−1^ Synacinn™ normalized the water intake, eliminating 91% of the increase. Additional increases in the water consumption of diabetic rats of 65%, 30%, and 26% were observed for those treated with 10, 50, and 100 mg kg^−1^ Synacinn™. Meanwhile, no changes in the water intake of diabetic rats treated with glibenclamide were observed.

#### 2.2.2 Bodyweight

STZ-induced diabetic rats are associated with body weight losses due to muscle wasting and the loss of tissue proteins.^28,29^ As listed in Table 1, after 28 days, the weights of the normal rats remain unchanged. In contrast, 22.3% weight reduction was observed in STZ-induced rats. Treatment with Synacinn™ at all concentrations led to the maintaining of the initial body weights of the diabetic rats. Insignificant changes (*p* > 0.05) were observed at doses of 10 mg kg^−1^ (3.5%), 50 mg kg^−1^ (−8.6%), 100 mg kg^−1^ (4.2%), 250 mg kg^−1^ (4.6%), and 250 (b.i.d.) mg kg^−1^ (5.2%). A notable weight gain in glibenclamide treated rats (11.2%) was measured, in agreement with previous reports.^30,31^

**Table 1:** The effects of Synacinn™ on body weights in normal and diabetic rats^a^

	Bodyweight		
	Initial body weight (g)	Final body weight (g)	% difference
Normal control	213.3 ± 11.4	215 ± 10.2	0.8^c^
Normal treated	223.3 ± 14.7	230 ± 12.1	3.0^c^
Diabetic control	216.7 ± 9.5	168.3 ± 6.5	−22.3^b^
D + 10 mg kg^−1^ Synacinn™	170 ± 7.3	176 ± 10.2	3.5^c^
D + 50 mg kg^−1^ Synacinn™	175 ± 16.5	160 ± 7.7	−8.6^c^
D + 100 mg kg^−1^ Synacinn™	190 ± 10.3	198 ± 11.1	4.2^c^
D + 250 mg kg^−1^ Synacinn™	181.7 ± 14.7	190 ± 7.7	4.6^c^
D + 250 (b.i.d.) mg kg^−1^ Synacinn™	232 ± 14.5	244.2 ± 14.7	5.2^c^
D + 6 mg kg^−1^ glibenclamide	193.3 ± 14.3	215 ± 9.9	11.2^c^

a Values are mean ± SEM; *n* = 6 rats per group.

b *p* < 0.05 compared with the initial body weight.

c *p* < 0.05 compared with the diabetic control.

#### 2.2.3 Relative organ weights and histological analysis

**Fig. 7:**
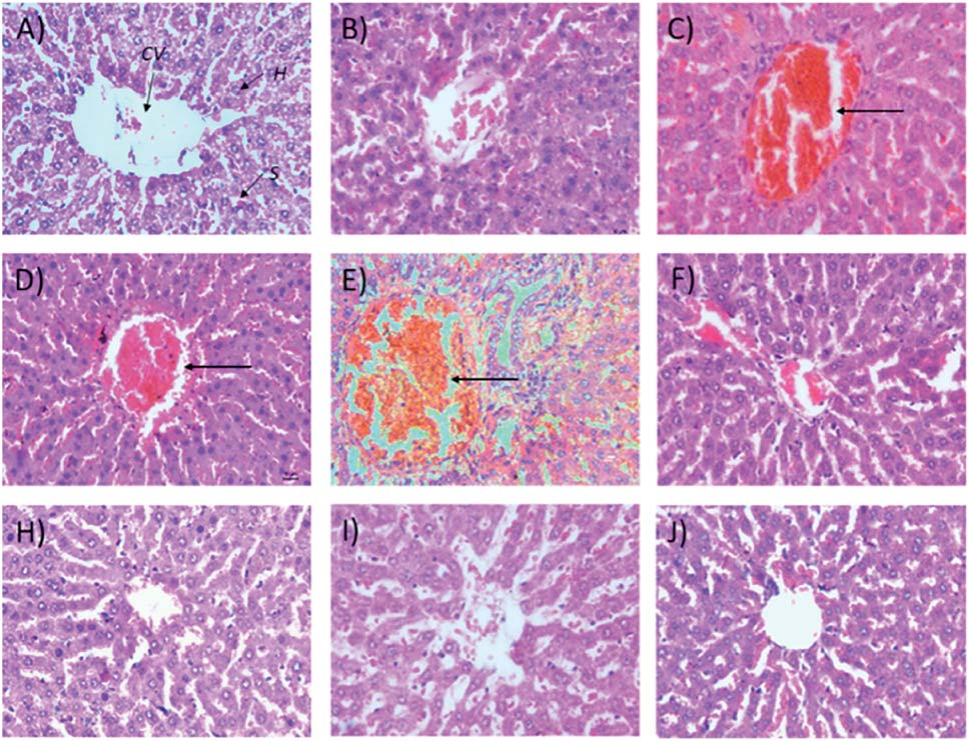
Histological structures of liver samples after 28 days of treatment (H&E, 40×). CV = central vein, H = hepatocytes, S = sinusoidal spaces.

Table 2 shows that the administration of STZ increases the relative weights of the liver (50%), kidney (39%), and pancreas (81%). Treatment with Synacinn™ significantly restores the relative weights of these organs to the normal non-diabetic values. The liver weights were reduced significantly (*p* < 0.05) after treatment with 250 mg kg^−1^ (31%) and 250 mg kg^−1^ (b.i.d.) Synacinn™ (33%) compared to the diabetic control. Histological analysis of normal control (Fig. 7A) and normal treated rats (Fig. 7B) showed a normal central vein surrounded by normal hepatocyte cells and sinusoidal spaces. The diabetic control rats (Fig. 7C) displayed noticeable congestion of the central vein, pathologically similar to the diabetic rats treated with 10 and 50 mg kg^−1^ Synacinn™ (Fig. 7C and D). Diabetic rats treated with 100 mg kg^−1^, 250 mg kg^−1^, and 250 (b.i.d.) mg kg^−1^ Synacinn™ and 6 mg kg^−1^ glibenclamide showed normalization of the hepatic structures with normal central vein and hepatocyte architectures.

**Table 2:** The effects of Synacinn™ on relative organ weights in normal and diabetic rats^a^

Treatment	Liver	Kidney	Pancreas
Normal control	3.61 ± 0.37	0.85 ± 0.08	0.32 ± 0.03^c^
Normal treated	3.67 ± 0.06^c^	0.72 ± 0.06^c^	0.31 ± 0.04^c^
Diabetic control	5.44 ± 0.21^b^	1.18 ± 0.04	0.58 ± 0.08^b^
D + 10 mg kg^−1^ Synacinn™	4.85 ± 0.39	1.12 ± 0.18	0.44 ± 0.04
D + 50 mg kg^−1^ Synacinn™	4.79 ± 0.23	0.82 ± 0.02^c^	0.38 ± 0.04^c^
D + 100 mg kg^−1^ Synacinn™	4.89 ± 0.42	0.92 ± 0.10	0.40 ± 0.04
D + 250 mg kg^−1^ Synacinn™	3.75 ± 0.27^c^	0.96 ± 0.13	0.34 ± 0.04^c^
D + 250 (b.i.d.) mg kg^−1^ Synacinn™	3.54 ± 0.26^c^	0.76 ± 0.07^c^	0.37 ± 0.02^c^
D + 6 mg kg^−1^ glibenclamide	3.88 ± 0.32^c^	0.96 ± 0.02	0.36 ± 0.01^c^

a Values are mean ± SEM; *n* = 6 rats per group.

b *p* < 0.05 compared to the normal control.

c *p* < 0.05 compared with the diabetic control group; one-way ANOVA followed by Tukey's test for *post-hoc* analysis. D = diabetic rats.

**Fig. 8:**
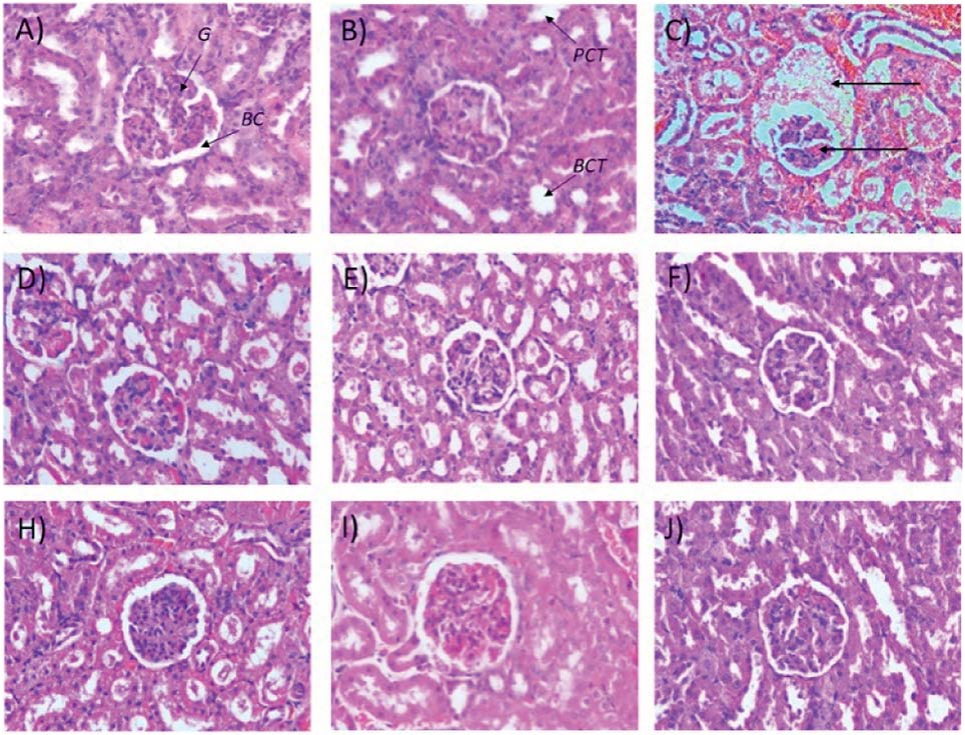
Histological structures of kidney samples after 28 days of treatment (H&E, 40×). G = glomerulus, BC = Bowman's capsule, PCT = proximal convoluted tubule, DCT = distal convoluted tubule.

It also was observed that 50 mg kg^−1^ and 250 (b.i.d.) mg kg^−1^ Synacinn™ restored the kidney weights to the normal state (31% and 36% decreases from the diabetic control, respectively). Histological analysis of the normal control (Fig. 8A) and normal rats treated with 250 mg kg^−1^ Synacinn™ (Fig. 8B) showed normal Bowman's capsule, glomerulus, and distal and proximal convoluted tubule structures. The enlargement of the Bowman's capsule and the degeneration of glomeruli were found in diabetic control rats (Fig. 8C). Meanwhile, diabetic rats treated with 10, 50, 100, 250, and 250 (b.i.d.) mg kg^−1^ Synacinn™ showed normal glomerulus features surrounded by a Bowman's capsule with normal distal and proximal convoluted tubules.

**Fig. 9:**
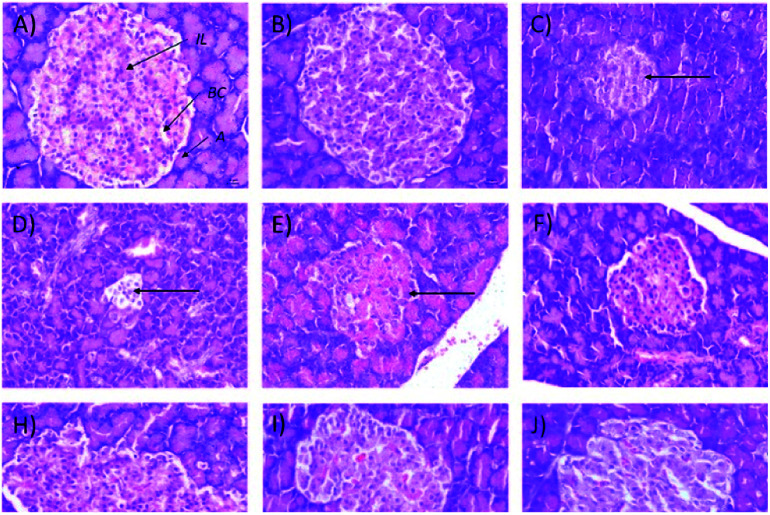
Histological structures of pancreas samples after 28 days of treatment (H&E, 40×). IL = islets of Langerhans, A = acinar cells, BC = blood capillary.

**Fig. 10:**
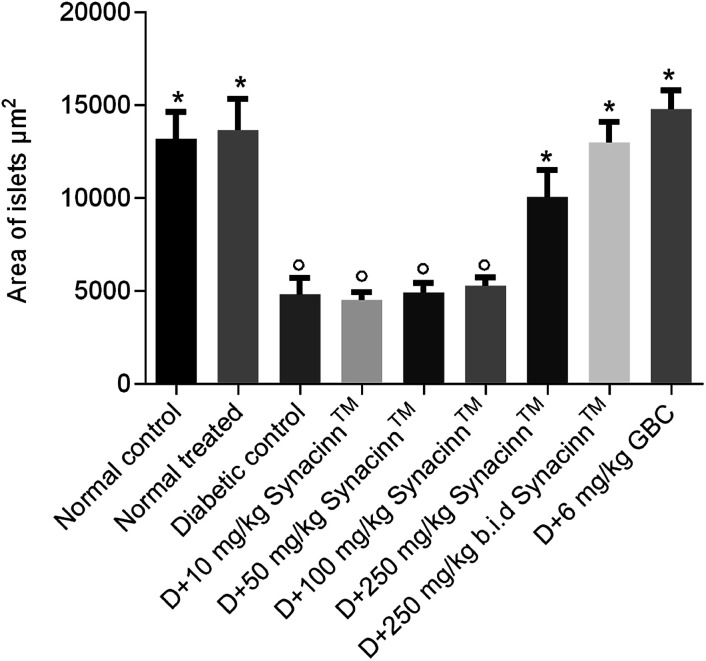
The effects of Synacinn™ on the islet areas. Values are mean ± SEM; *n* = 6 rats per group; °: *p* < 0.05 as compared to the normal control; *: *p* < 0.05 compared with the diabetic control group; one-way ANOVA followed by Dunnett's test for *post-hoc* analysis. D = diabetic rats, GBC = glibenclamide.

In addition, the relative pancreas weights also improved compared with the diabetic control when treated with 50 mg kg^−1^ (35% decrease), 250 mg kg^−1^ (42% decrease), and 250 (b.i.d.) mg kg^−1^ Synacinn™ (36% decrease). Histological structures displayed normal pancreatic tissue with well-demarcated islets of Langerhans and acinar cells in the normal control rats (Fig. 9A) and normal rats treated with 250 mg kg^−1^ Synacinn™ (Fig. 9B). Meanwhile, the pancreatic tissue of the diabetic rats (Fig. 9C) displayed visible morphological changes. Histopathological analysis of diabetic control rats and diabetic rats treated with 10, 50, and 100 mg kg^−1^ Synacinn™ showed a reduction in the size of the islets and damage to the β-cell population of the pancreatic tissue. The treatment of diabetic rats with 250 and 250 (b.i.d.) mg kg^−1^ Synacinn™ and 6 mg kg^−1^ glibenclamide improved the pancreatic tissue, increasing the size of the islets and the number of pancreatic β-cells to near normal control levels. As seen in Fig. 10, diabetic control rats and diabetic rats treated with 10, 50, and 100 mg kg^−1^ Synacinn™ showed a significant reduction (*p* < 0.05) in the area of the islets compared to the normal control rats. However, treatment with 250 and 250 (b.i.d.) mg kg^−1^ Synacinn™ and 6 mg kg^−1^ glibenclamide could improve the condition of the pancreatic islets, significantly increasing (*p* < 0.05) the area of the islets when compared to the diabetic control rats. The results suggest that Synacinn™ acted as a hepaprotective, renoprotective and pancreas-regenerative agent in STZ-induced rats.

#### 2.2.4 Fasting blood glucose, total cholesterol, and triglyceride levels

The treatment with Synacinn™ of STZ-induced rats for 28 days significantly improved fasting blood glucose, triglyceride, and cholesterol levels in the blood. As referred to in Table 3, only 250 mg kg^−1^ (b.i.d.) Synacinn™ normalized the fasting blood glucose level as early as day 7, and it continued to maintain normal levels until the end of the experiment, 59% lower compared to the diabetic control. On day 28, there were notable reductions in the blood glucose levels of diabetic rats treated with 10 mg kg^−1^ (4.7%), 50 mg kg^−1^ (29.1%), 100 mg kg^−1^ (41.2%), and 250 mg kg^−1^ (37.5%) Synacinn™, but they were still within the hyperglycemic range. Meanwhile, the treatment of normal rats with 250 mg kg^−1^ Synacinn™ did not have any hypoglycemic effect.

**Table 3:** The effects of Synacinn™ on fasting blood glucose levels in normal and diabetic rats. D = diabetic rats

Group	Fasting blood glucose (mmol L^−1^)				
	0^th^ day	7^th^ day	14^th^ day	21^st^ day	28^th^ day
Normal control	10.35 ± 0.7	9.73 ± 0.5	9.97 ± 0.4	11.18 ± 0.6	9.38 ± 0.5^b^
Normal treated	10.36 ± 0.6	7.93 ± 0.5	7.83 ± 0.3^b^	9.4 ± 0.2	9.2 ± 1.4^b^
Diabetic control	16.25 ± 2.0	19.95 ± 1.5	23.28 ± 2.3	21.43 ± 2.2	27.93 ± 2.6^a^
D + 10 mg kg^−1^ Synacinn™	18.95 ± 1.9^a^	17.28 ± 2.4	29.7 ± 2.7^a^	29.5 ± 3.6^a^	26.62 ± 1.1^a^
D + 50 mg kg^−1^ Synacinn™	17.55 ± 1.8	17.7 ± 2.5	21.87 ± 4.3	24.22 ± 4.2	19.78 ± 4.8
D + 100 mg kg^−1^ Synacinn™	12.70 ± 2.4	19.03 ± 3.1	29.62 ± 3.9^a^	24.42 ± 5.0	16.43 ± 4.2
D + 250 mg kg^−1^ Synacinn™	15.08 ± 1.4	22.3 ± 3.3^a^	15.08 ± 2.0	20.2 ± 3.1	17.47 ± 2.0
D + 250 (b.i.d.) mg kg^−1^ Synacinn™	14.82 ± 1.3	8.25 ± 1.7^b^	9.8 ± 0.39	8.52 ± 0.5	11.57 ± 0.4^b^
D + 6 mg kg^−1^ glibenclamide	19.77 ± 4.1^a^	11.7 ± 2.6	15.77 ± 5.42	12.17 ± 4.1	18.2 ± 4.3

a *p* < 0.05 as compared to normal control.

b *p* < 0.05 compared with the diabetic control group.

As referred to in Table 4, the cholesterol level was increased by 28% in STZ-induced diabetic rats. This increase was diminished upon Synacinn™ treatment at all tested concentrations. Cholesterol level reductions of 34%, 44%, 63%, 34%, and 47% were measured upon treatment with 10, 50, 100, 250, and 250 (b.i.d.) mg kg^−1^ Synacinn™. Moreover, the cholesterol level in 100 mg kg^−1^ treated rats was significantly (*p* < 0.05) reduced to a level below the normal control rat level.

**Table 4:** The effects of Synacinn™ on cholesterol and triglyceride levels in normal and diabetic rats

Group	Cholesterol (mmol L^−1^)	Triglyceride (mmol L^−1^)
Normal control	2.5 ± 0.2	1.0 ± 0.1
Normal treated	2.5 ± 0.2	1.0 ± 0.2
Diabetic control	3.2 ± 0.2	1.9 ± 0.3
D + 10 mg kg^−1^ Synacinn™	2.1 ± 0.3^b^	1.3 ± 0.3
D + 50 mg kg^−1^ Synacinn™	1.8 ± 0.2^b^	1.7 ± 0.3
D + 100 mg kg^−1^ Synacinn™	1.2 ± 0.2^a^^,^^b^	0.4 ± 0.1^b^
D + 250 mg kg^−1^ Synacinn™	2.1 ± 0.2^b^	1.3 ± 0.1
D + 250 (b.i.d.) mg kg^−1^ Synacinn™	1.7 ± 0.1^b^	0.8 ± 0.1^b^
D + 6 mg kg^−1^ glibenclamide	1.7 ± 0.1^b^	1.0 ± 0.3

a *p* < 0.05 as compared to normal control.

b *p* < 0.05 compared with the diabetic control group.

For the triglyceride levels (Table 4), a 90% increase was detected in diabetic control rats. A notable reduction was measured after treatment with 10 mg kg^−1^ (32%), 50 mg kg^−1^ (11%), and 250 mg kg^−1^ (32%) Synacinn™. However, treatment with 100 and 250 mg kg^−1^ (b.i.d.) Synacinn™ significantly (*p* < 0.05) reduced triglyceride levels by 79% and 58% when compared to the diabetic control rats.

#### 2.2.5 Liver and renal function

**Fig. 11:**
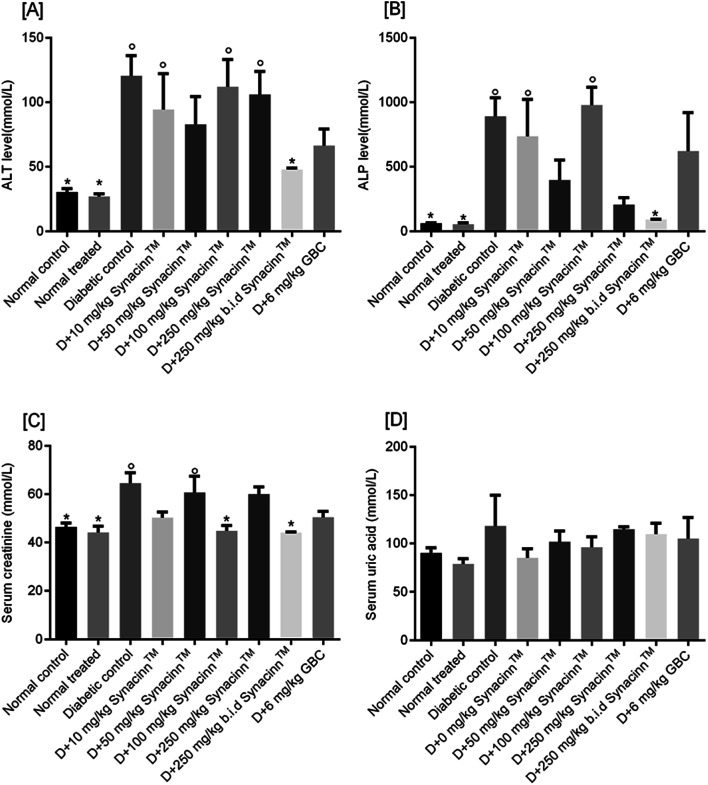
The effects of Synacinn™ on (A) liver ALT, (B) liver ALP, (C) creatinine, and (D) uric acid levels. Values are mean ± SEM; *n* = 6 rats per group; °: *p* < 0.05 compared to the normal control; *: *p* < 0.05 compared with the diabetic control group; one-way ANOVA followed by Dunnett's test for *post-hoc* analysis. GBC = glibenclamide.

The hyperglycemic condition of STZ-induced diabetic rats elevates the levels of liver dysfunction proteins, such as alanine aminotransferase (ALT) and alkaline phosphatase (ALP).^32^ As shown in Fig. 11A, the ALT levels of STZ-induced diabetic control rats were increased three-fold as compared to the normal control. Furthermore, treatment with 10, 50, 100, and 250 mg kg^−1^ Synacinn™ was unable to reduce the ALT levels back to the normal level. Interestingly, 250 (b.i.d.) mg kg^−1^ Synacinn™ resulted in significant (*p* < 0.05) recovery of liver function, with the ALT level decreasing by 60% when compared to the diabetic control rats.

Meanwhile, for ALP, as presented in Fig. 11(B), a 13-fold increase was measured in the STZ-induced diabetic control as compared to the normal control. Treatment with 10, 50, and 100 mg kg^−1^ Synacinn™ failed to reduce the ALP levels in the blood serum of diabetic rats. Significant reductions in ALP levels were observed in diabetic rats treated with 250 mg kg^−1^ Synacinn™ and 250 (b.i.d.) mg kg^−1^ Synacinn™, 77% and 90% lower than the diabetic control. Surprisingly, Synacinn™ performs better than the positive control. Notable reductions in ALT and ALP levels in diabetic rats treated with glibenclamide were seen, but these were insignificant compared to the diabetic control.

Diabetic nephropathy is detected based on elevated creatinine and uric acid levels in the blood. In this study, the serum creatinine level was significantly (*p* < 0.05) increased by around 40% compared to the normal control. Treatment with Synacinn™ at 100 and 250 (b.i.d.) mg kg^−1^ significantly (*p* < 0.05) reduced the creatine levels to normal levels (31% and 32% decreases), better than glibenclamide (22% decrease). Meanwhile, the uric acid levels of diabetic rats are slightly higher compared to the normal rats. However, no significant (*p* > 0.05) differences are observed for all diabetic rats when compared to the normal rats.

#### 2.2.6 HPLC analysis

**Fig. 12:**
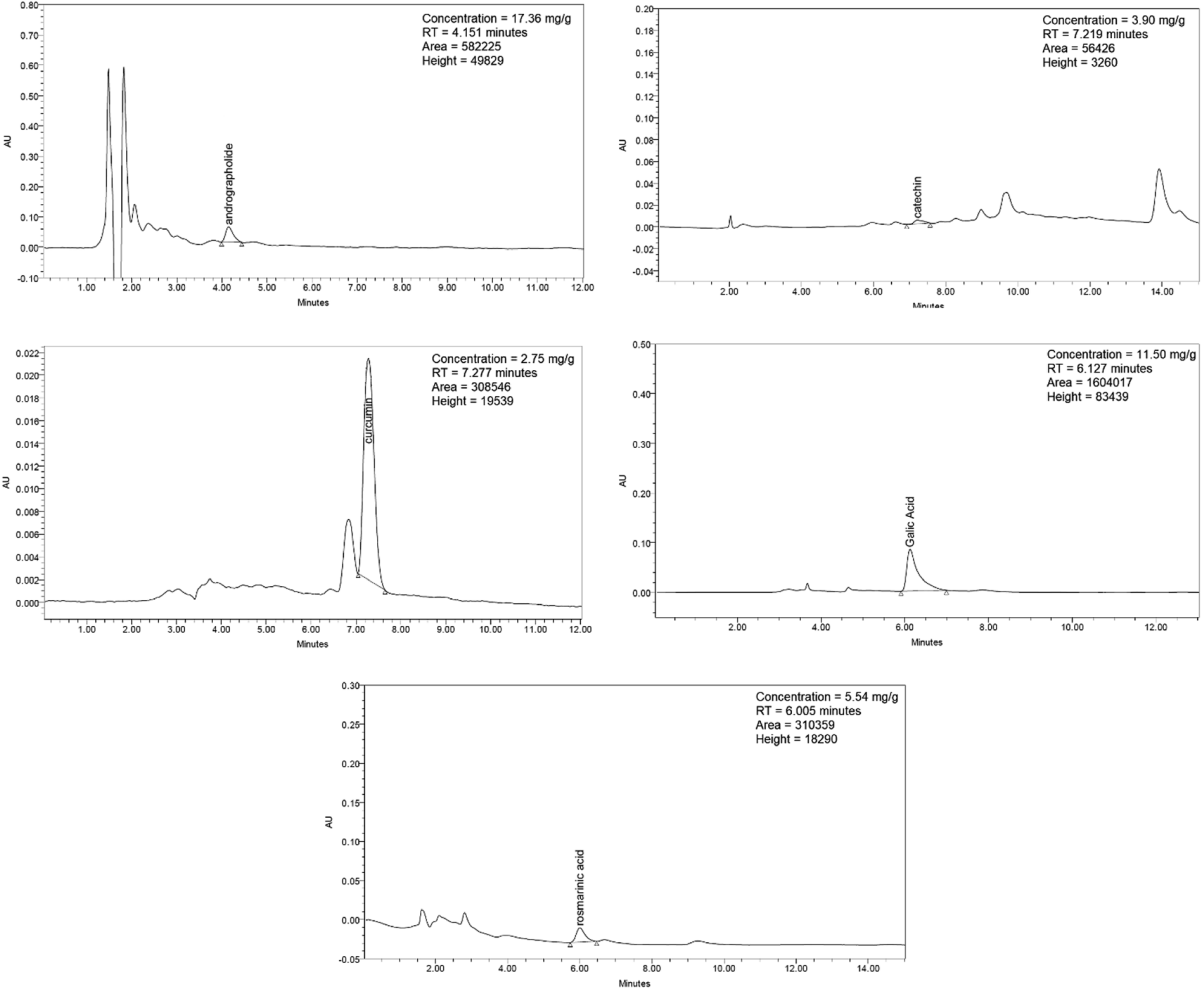
HPLC chromatograms of andrographolide, catechin, curcumin, gallic acid, and rosmarinic acid.

Five phytochemicals were selected, andrographolide, curcumin, catechin, gallic acid, and rosmarinic acid, as previously detected in *Andrographis paniculata* (Burm.f.) Nees,^33^*Curcuma zanthorrhiza* Roxb.,^34^*Cinnamomum zeylanicum* Blume,^35^*Syzygium polyanthum* (Wight) Walp,^36^ and *Orthosiphon stamineus* Benth.,^37^ respectively. The phytochemical concentrations were 17.36 μg mg^−1^ for andrographolide, 2.75 μg mg^−1^ for curcumin, 3.90 μg mg^−1^ for catechin, 11.50 μg mg^−1^ for gallic acid, and 5.54 μg mg^−1^ for rosmarinic acid. The HPLC chromatograms for each phytochemical are illustrated in Fig. 12.

#### 2.2.7 *In silico* interactions of the active phytochemicals with glucokinase

**Fig. 13:**
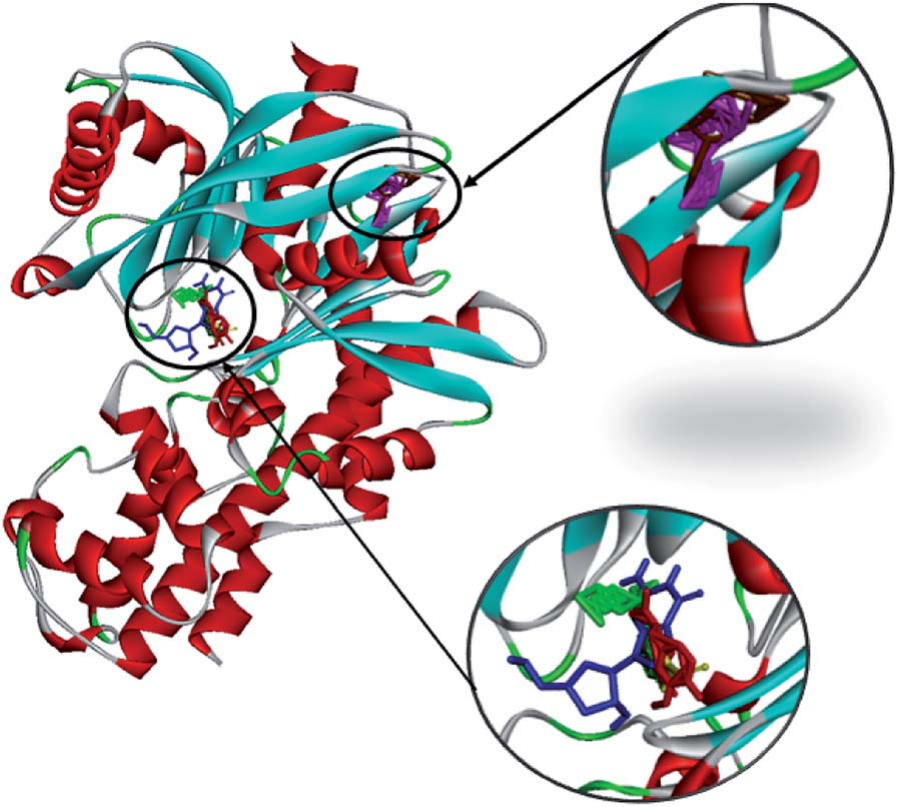
The molecular docking of andrographolide (magenta), catechin (brown), curcumin (green), gallic acid (blue), and rosmarinic acid (red) with 1V4S.

**Fig. 14:**
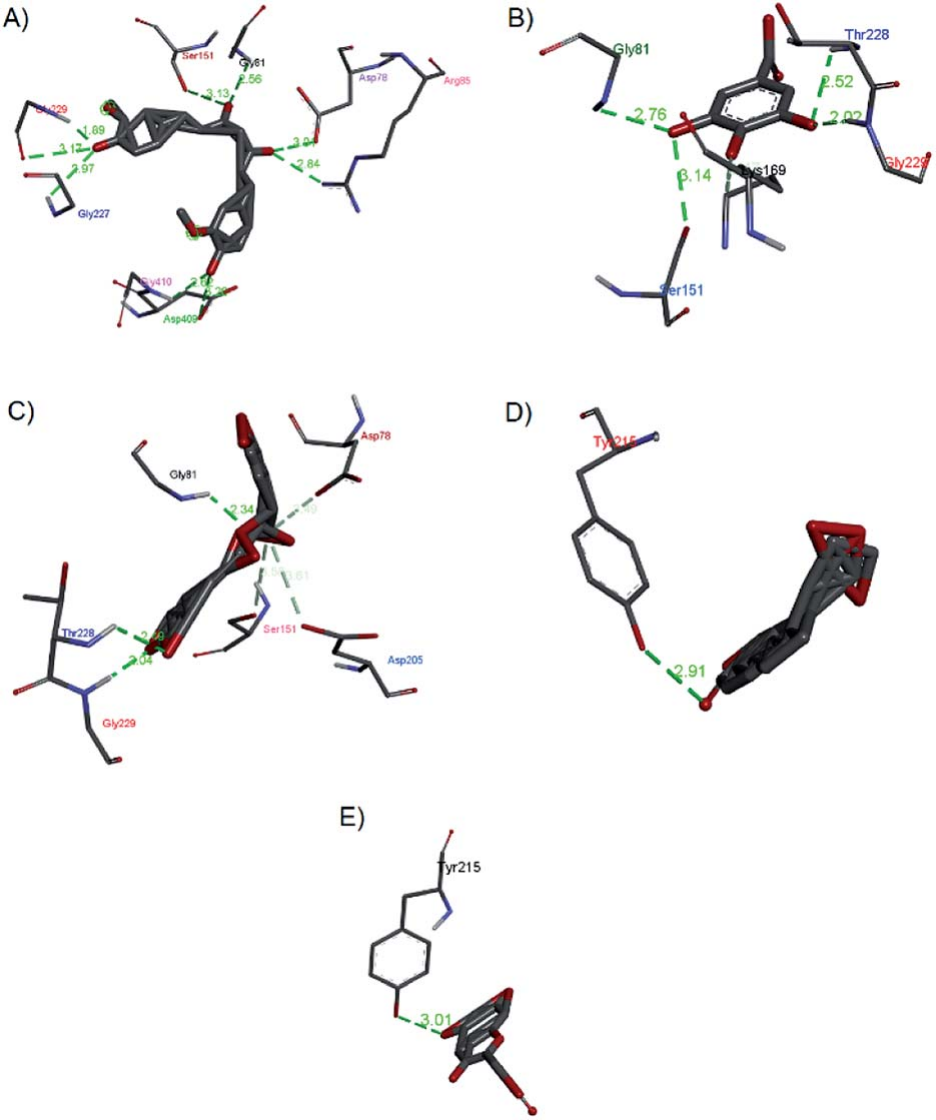
Hydrogen bond interactions of (a) curcumin, (b) gallic acid, (c) rosmarinic acid, (d) andrographolide, and (e) catechin with 1V4S.

**Fig. 15:**
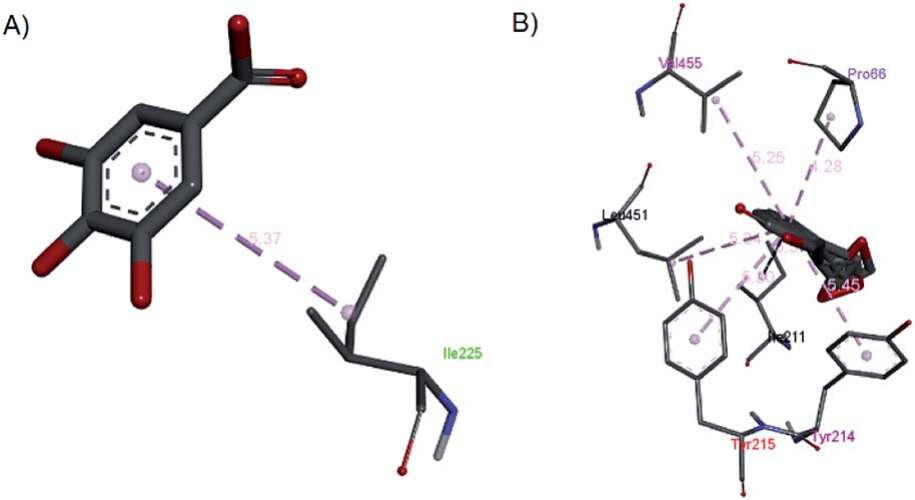
The hydrophobic contacts of (a) gallic acid and (b) andrographolide with 1V4S.

Investigations into the binding activities of andrographolide, curcumin, catechin, gallic acid, and rosmarinic acid with human glucokinase protein (PDB ID: 1V4S) were carried out using a molecular docking approach. The binding affinity was highest for andrographolide (−12.1 kcal mol^−1^), followed by catechin (−10.2 kcal mol^−1^), rosmarinic acid (−8.6 kcal mol^−1^), curcumin (−7.8 kcal mol^−1^), and gallic acid (−5.6 kcal mol^−1^). The docked conformations showed two different binding site locations (Fig. 13), with andrographolide and catechin docked in the same binding region, and gallic acid, rosmarinic acid, and curcumin binding in the other region. Fig. 14 and 15 illustrate the hydrogen bond interactions and hydrophobic contacts of these compounds. In this study, the docking of andrographolide with 1V4S showed that hydrogen bond interactions were established with Tyr215 (2.34 Å) (Fig. 14D), while Tyr215, Tyr214, Val455, Pro66, Leu451, and Ile211 (Fig. 15B) formed strong hydrophobic contacts with interatomic distances of 5.30 Å, 5.45 Å, 5.25 Å, 4.28 Å, 5.34 Å, and 3.30 Å, respectively. Hydrophobic contact residues were observed in the interactions of quinazoline-4-one derivatives with glucokinase activators.^38^ These interactions fit well in the binding pocket of 14VS. The docked conformation of rosmarinic acid (Fig. 14C) with 1V45 showed that Gly81, Gly229, Thr228, Asp78, Asp205, and Ser151 form strong hydrogen bond interactions with interatomic distances that are 2.34 Å, 2.09 Å, 2.49 Å, 3.04 Å, 3.09 Å, and 3.51 Å, respectively, from the receptor. Similar amino acid interactions, such as those involving glycine and tyrosine, are seen in the inhibition of type 2 diabetes mellitus.^39^ The docking of curcumin (Fig. 14A) with the receptor resulted in the highest number of hydrogen bonds, involving Gly229, Gly227, Gly410, Gly81, Asp409, Asp78, Arg85, and Ser151 with interatomic distances that are 1.86 Å, 2.97 Å, 2.62 Å, 2.56 Å, 3.02 Å, 3.01 Å, 2.84 Å, and 3.31 Å, respectively, from the receptor. Similar interactions were also observed in curcumin inhibition studies towards PPAR-gamma.^40^

Gallic acid showed hydrogen bond interactions (Fig. 14B) with Gly81, Gly229, Ser151, Thr228, and Lys169 residues and a hydrophobic interaction with Ile25. The ring of gallic acid forms a strong interaction with a hydrophobic residue (isoleucine), and two hydrogen bonds also form with hydrophobic residues (tyrosine and serine). These interactions might contribute to the stability of the compound in pharmacokinetics studies, in which this compound remains stable for a few hours after intake. The docking of catechin (−10.2 kcal mol^−1^) and andrographolide (−12.1 kcal mol^−1^) with 1V4S resulted in the most stable binding energies, however hydrogen bond interactions with only one residue, Tyr215, were observed with interatomic distances of 3.01 Å and 2.91 Å, respectively, from the receptor (Fig. 14D and E). In this case, catechin uses the aromatic ring to interact with tyrosine in the catalytic cavity, providing strong hydrogen bond interactions. These data are consistent with a study that found that the interaction of tyrosine with α-amylase affects the glucose metabolism and that tyrosine was located at the active sites of the enzymes.^41^ Other findings suggest that the galloyl moiety in catechin binds to PPA and promotes the entering of polyphenol and association with the active site of the enzyme.^42^ The high stability of catechin in the 1V4S protein might be related to interactions with tyrosine at the active site of the enzyme.

## 3. Discussion

Evaluation of toxicity and efficacy using animal models is necessary to demonstrate the desired pharmacological effects of treatments with drug candidates. In our acute toxicity tests, The LD_50_ value of Synacinn™ was found to be above 2000 mg kg^−1^, which, according to the GHS, means it is graded as class 5. These results suggest that, upon treatment with a single limited dose, Synacinn™ has a wide margin of safety. In addition, Organization for Economic Co-operation and Development (OECD) recommendations for acute oral toxicity list an LD_50_ value equivalent to 2000 mg kg^−1^ or above as “unclassified”, indicating that Synacinn™ is considerably safe to consume.

High doses of plant extracts can be metabolized into toxic end products, which may affect gastric function and reduce food conversion efficiency.^43,44^ In the 14 day repeated oral dose toxicity study, Synacinn™ did not affect the intake of food and water, even at the highest dose (2000 mg kg^−1^), particularly during the administration period. Food and water were suitably accepted by the rats treated at all doses of Synacinn™, suggesting that this polyherbal formulation may not cause any alterations to protein, carbohydrate, and fat metabolisms. Also, Synacinn™ did not adversely affect nutritional aspects such as appetite stability and weight gain, which remained at the normal levels for animals that are constantly supplied with food and water *ad libitum*. Therefore, Synacinn™ at the tested doses can be considered to be non-toxic in terms of food consumption and water intake over the administration period of 14 days and the 14 day recovery period. From histopathological analysis, there were several signs of toxicity in livers and kidneys from rats treated with 600 and 2000 mg kg^−1^ Synacinn™ during both periods of study. Meanwhile, rats treated with 250 mg kg^−1^ Synacinn™ demonstrated normal kidney and liver structures during both periods of study. Since a 250 mg kg^−1^ dose level of Synacinn™ did not result in any significant effects on food and water intake, body weight changes, and relative organ weights, and resulted in no abnormalities upon a histopathological examination of livers and kidneys, this dose is considered as a safe oral dose and was used for anti-hyperglycemic efficacy testing.

In the efficacy study, type 1 diabetic rats induced by STZ were used to assess the therapeutic effects of Synacinn™ in the management of diabetes mellitus. Treatment with Synacinn™ was effective in reducing food consumption and significantly improving the typical symptoms of polyphagia in diabetic rats. Aside from this, food consumption in normal rats treated with 250 mg kg^−1^ Synacinn™ does not increase, and appetites are not suppressed. Interestingly, all tested doses of Synacinn™ possess comparable anti-hyperglycemic activities with the synthetic drug (glibenclamide) in terms of food-intake control. The satiety and feeding centers located in the ventromedial and lateral hypothalamus are a dual mechanism that plays an essential role in appetite regulation.^45^ The glucostatic regulation suggests the presence of glucoreceptors in these regions of the hypothalamus, which are sensitive to blood glucose utilization. The measurement of blood glucose utilization is carried out based on the arteriovenous glucose difference.^46^ If the difference is high due to greater glucose utilization by the satiety center, the satiety center will be activated, and if the difference is low, then the feeding center will be activated. In the case of diabetes mellitus, glucose cannot pass into the cells of the satiety center, thereby resulting in a low arteriovenous difference. This disorder stimulates the feeding center chronically and results in the existence of polyphagia.^46,47^ In addition, diabetic rats treated with 250 (b.i.d.) mg kg^−1^ Synacinn™ were found to substantially decrease their water intake relative to diabetic control rats. Since water intake is affected by osmoregulators that detect fluid osmolality, Synacinn™ at this concentration can lessen polydipsia symptoms in diabetic rats *via* inhibiting the anterior hypothalamus thirst core.

Insulin is a hormone that helps the transportation of glucose to adipocytes and muscle cells, increases the synthesis and storage of muscle glycogen, cellular proteins, and triglycerides in adipocytes, and reduces protein catabolism.^48,49^ Insulin deficiency leads to the inability of cells to utilize glucose for energy production and will cause the overstimulation of gluconeogenesis. Consequently, the use of muscle protein and exaggerated fat mobilization from adipose tissue for energy production during gluconeogenesis cause weight loss as a result of diabetes mellitus. Additionally, glucosuria is also reported to cause substantial calory loss for every gram of glucose excreted in urine. When combined with the loss of muscle and adipose tissue, this results in significant weight loss despite an increase in appetite.^47^ Hence, this clarifies the severe weight loss in the diabetic control rats, despite them having the highest food intake. Significant weight gain arose in the diabetic rats treated with Synacinn™, suggesting a preventive mechanism against muscle wasting and loss of tissue protein due to hyperglycemic conditions. Interestingly, Synacinn™ also exhibited a superior effect for restoring body weight loss compared to the synthetic drug (glibenclamide).

Anti-hyperglycemic monitoring during treatment with 250 mg kg^−1^ (b.i.d.) Synacinn™ demonstrated a significant lowering of fasting blood glucose levels. Moreover, the anti-hyperglycemic effect was detected as early as day 7, and levels remained consistent within a normal range throughout the treatment period. Multiple studies have reported anti-hyperglycemic activities during *in vivo* models for each herb used in the Synacinn™ formulation. Lower blood glucose levels in diabetic rats have been reported for *Cinnamomum zeylanicum* Blume,^7,8^*Orthosiphon stamineus* Benth.,^11^*Curcuma zanthorrhiza* Roxb.,^15^*Syzygium polyanthum* (Wight) Walp,^22^ and *Andrographis paniculate* (Burm.f.) Nees.^23^

At the cellular level, *Cinnamomum zeylanicum* Blume enhanced glucose uptake in 3T3-L1 adipocytes^50^ and C2C12 myotubes.^51^ Curcumin, a phytochemical from *Curcuma zanthorrhiza* Roxb., which is present in Synacinn™, not only enhances cellular glucose uptake^52^ but it also enhances the production of insulin in β-Min6 pancreatic beta cells.^53^ In addition, curcumin can inhibit hepatic gluconeogenesis, which in turn regulates the whole-body glucose metabolism.^54^ An aqueous extract of *Orthosiphon stamineus* (Burm.f.) Nees was found to stimulate glucose-induced insulin secretion in rat pancreases, which in turn effectively decreased the plasma glucose concentration in STZ-induced diabetic rats.^14^ Gallic acid in *Syzygium polyanthum* (Wight) Walp regulates the anti-diabetic activity through the inhibition of alpha-glucosidase and the lowering of the blood glucose level.^36^ Based on previous findings, it is suggested that the bioactive compounds present in Synacinn™ act synergistically to reduce the fasting blood glucose levels in STZ-induced diabetic rats through two mechanisms: increasing glucose uptake and utilization, and stimulating the secretion of insulin from pancreatic β-cells.

In addition to its glucose-lowering activity, the hypolipidemic effect of Synacinn™ is in accordance with earlier studies, which reported that *Cinnamomum zeylanicum* Blume,^10^*Curcuma zanthorrhiza* Roxb.,^17^*Andrographis paniculata* (Burm.f.),^11^*Orthosiphon stamineus* Benth.,^14^ and *Syzygium polyanthum* (Wight) Walp^21^ could lower serum triglyceride and cholesterol levels in animal models. Under normal conditions, insulin activates lipoprotein lipase to break down triglycerides. Ineffective insulin function, either due to a lack of insulin or/and insulin resistance, leads to failure during the activation of this enzyme, resulting in hypertriglyceridemia.^55,56^ The significant control of serum triglyceride levels in diabetic rats treated with Synacinn™ might be attributed to an enhancement in insulin levels or the insulin-like activity of this polyherbal formulation. Therefore, Synacinn™ is believed to possess anti-hyperglycemic activity, lowering the serum triglyceride and cholesterol levels in STZ-induced diabetic rats.

Besides the disruption of glucose metabolism activity, STZ administration leads to histopathological changes in most tissue samples, including liver samples.^57^ Our findings suggest that STZ-induced rats display liver hypertrophy with enlarged and inflamed hepatocytes due to an increased influx of fatty acids into the liver, leading to the overproduction and accumulation of intracellular triglycerides.^58,59^ Meanwhile, normal treated rats and diabetic rats treated with 250 and 250 (b.i.d.) mg kg^−1^ Synacinn™ and 6 mg kg^−1^ glibenclamide showed significantly (*p* < 0.05) lower relative liver weights when compared to the diabetic control rats, indicating the ameliorating effects of Synacinn™ on liver hypertrophy. The liver structure of STZ-induced diabetic rats showed a congested central vein and the thickening of the wall of the vein, which is in agreement with previous reports.^60,61^ The treatment of diabetic rats with Synacinn™ to some extent protected them from these changes and preserved the liver architecture in a similar fashion to normal control rats. Additionally, our data showed that the induction of diabetes in rats *via* STZ administration clearly caused higher ALT and ALP levels, and treatment with 250 mg kg^−1^ (b.i.d.) Synacinn™ normalized the levels by 60% and 90%. Elevated ALT activity is associated with a loss of the functional integrity of the cell membrane in the liver.^62^ Meanwhile, elevated hepatic ALP levels indicate cholestasis (failure of bile flow) rather than simple damage to liver cells.^63^ Therefore, these findings suggest that Synacinn™ might possess a hepatoprotective effect through protection against hepatocellular injury and cholestasis.

Diabetic nephropathy is a diabetes mellitus microvascular complication that can lead to end-stage renal disease.^64^ In our study, notably increased relative kidney weights in STZ-induced diabetic rats might be due to hypertrophic glomerular conditions, glomerulosclerosis, tubulointerstitial inflammation, and fibrosis. Treatment with 250 mg kg^−1^ (b.i.d.) Synacinn™ significantly reduced the inflamed kidney weight and restored the enlarged kidney Bowman's capsules and glomerulus degeneration to the normal state. Measurements of uric acid and creatinine levels are used to diagnose or monitor acute and chronic renal disease.^65^ Our data showed that uric acid levels are not affected by STZ induction. However, elevated levels of creatinine in STZ-induced rats are detected and treatment with Synacinn™ normalizes them. Creatinine is a metabolite derived from creatine, which is produced by the liver and transported to muscles. It is commonly produced at a constant rate and usually eliminated from the blood by the renal system. Therefore, elevated levels of creatinine indicate the occurrence of renal injury.^66^ The administration of Synacinn™ at 100 and 250 (b.i.d.) mg kg^−1^ for 28 days causes a significant (*p* < 0.05) reduction of creatinine levels. This result suggests that this polyherbal formulation could improve the general condition of diabetic rats and is able to restore renal function to near-normal conditions. This result agrees with previous investigation of the individual herbs in Synacinn™, where it was found that *Cinnamomum zeylanicum* Blume,^10^*Andrographis paniculata* (Burm.f.),^25^ and *Orthosiphon stamineus* Benth.^67^ could reduce increased serum creatinine levels in diabetic rats. Even though there has been limited research into *Curcuma zanthorrhiza* Roxb. and *Syzygium polyanthum* (Wight) Walp, findings relating to the renal protective effects of their bioactive compounds, such as curcumin and gallic acid, have been reported. Curcumin minimized increased kidney function marker levels (creatinine and urea) in STZ-induced diabetic rats.^68^ Meanwhile, elevated plasma creatinine levels during type 1 diabetes mellitus are reduced upon treatment with gallic acid.^69^ Hence, the herbs in Synacinn™ might act individually or synergistically to reduce elevated serum creatinine levels in STZ-induced diabetic rats.

STZ is a potent methylating agent and it is widely known to be able to deteriorate pancreatic β-cells *via* breaking DNA strands, consequently leading to insufficient insulin levels in the blood.^70^ The results show that pancreas weights are significantly increased in STZ-induced rats, suggesting the occurrence of inflammation or edema in this organ.^71^ Upon treatment with Synacinn™, the weights of the inflamed pancreases were reduced by almost 50%, indicating the existence of a protection process and regeneration mechanism. Histopathology analysis revealed that STZ-induced diabetic rats have small and shrunken islets, along with the destruction of the β-cell population in the endocrine part of the pancreas tissue. Treatment with 250 and 250 (b.i.d.) mg kg^−1^ Synacinn™ and 6 mg kg^−1^ glibenclamide for 28 days could lessen or regenerate the condition of the islets when compared to the diabetic control rats. These findings suggest that treatment with Synacinn™ might protect islet cells from morphological damage in STZ-induced diabetic rats, comparable with synthetic drugs, and this could offer new hope for DM patients in the future.

Healthy pancreases and livers secrete a highly localized enzyme, which is vital for proper glucose metabolism. Glucokinase plays a role as a glucose-controlling sensor in pancreatic β-cells, directly determining the insulin secretion levels. In the liver, glucokinase is involved in the conversion of glucose to glycogen. Through *in silico* modeling, five phytochemicals in Synacinn™ are shown to bind to this human glucokinase protein, with andrographolide and catechin showing the highest binding affinities. The interaction complexes between these phytochemicals and glucokinase could enhance the enzyme activity in a specific area, thus producing anti-hyperglycemic activity.

## 4. Experimental

### 4.1 Materials

Hydrochloric acid, xylene, acetic acid, glycerol, ethanol, formaldehyde, trisodium citrate dihydrate, and citric acid monohydrate were purchased from Merck (Selangor, Malaysia). Potassium acetate, Wright's stain, and paraffin plasticized pellets were obtained from BDH (Leicestershire, England). Streptozotocin (STZ) (98%, HPLC), Harris hematoxylin solution, and eosin Y were purchased from Sigma-Aldrich (Selangor, Malaysia). Glibenclamide was purchased from Pharmaniaga (Selangor, Malaysia). Synacinn™, with the registration number MAL16040030TC, was supplied by Proliv Life Sciences (Malaysia).

### 4.2 Toxicity studies

#### 4.2.1 Acute toxicity studies

Healthy female Sprague-Dawley rats (120–160 g, 6–8 weeks) were purchased from Takrif Bistari Enterprise (Selangor, Malaysia). They were housed in cages at ambient temperature and provided with a lighting period of 12 hours daily at relative humidity of 50–60%. The rats were fed with a standard pellet diet and had free access to water *ad libitum*. They were allowed to acclimatize for seven days prior to the experiments. The experimental protocol was approved by the Ethics Committee of Universiti Malaysia Terengganu with the registration number UMT/JKEPHT/2017/3. Acute toxicity studies were conducted following OECD 420: Acute Oral Toxicity – Fixed Dose Procedure, 2001. The animals were randomly assigned into two groups of six animals per group and received the following treatments:

Group 1: normal rats received distilled water.

Group 2: normal rats received 2000 mg kg^−1^ Synacinn™.

#### 4.2.2 14 day repeated oral dose toxicity studies

Twenty-four female Sprague-Dawley rats were randomly assigned to four groups with six animals per group and received the following treatments:

Group 1: normal rats received distilled water (control).

Group 2: normal rats received 250 mg kg^−1^ Synacinn™.

Group 3: normal rats received 600 mg kg^−1^ Synacinn™.

Group 4: normal rats received 2000 mg kg^−1^ Synacinn™.

Samples (1 mL) were given orally for 14 days. Food consumption, water intake, and body weight gains were recorded on days 0, 3, 7, 14, 21, and 28 during treatment. Blood was drawn from the tail vein on days 0, 7, 14, 21, and 28. Half of the surviving rats (*n* = 3) from each group were sacrificed on day 14 of the repeated dose study to obtain organs such as livers and kidneys to assess their relative weights. Half of the surviving rats (*n* = 3) were kept for another 14 days as an observation period. Histological examinations were conducted to detect any abnormalities and toxicological signs.

### 4.3 Efficacy studies

#### 4.3.1 Induction of diabetes

The rats fasted overnight (12–16 hours) prior to diabetes induction but were allowed free access to water *ad libitum*. Hyperglycemic conditions were induced *via* a single intraperitoneal injection of freshly prepared STZ (60 mg kg^−1^ body weight) in 0.1 M citrate buffer (pH: 4.5). Control groups were treated with citrate buffer (pH: 4.5). After seven days, fasting blood glucose levels were determined, and rats with a level ≥11.8 mmol L^−1^ were considered diabetic and used for these experiments.

#### 4.3.2 Animal grouping

The animals were randomly assigned into nine groups with six animals per group and received the following treatments:

Group 1: normal rats treated with the vehicle (distilled water).

Group 2: normal rats treated with 250 mg kg^−1^ Synacinn™.

Group 3: diabetic rats treated with the vehicle (distilled water).

Group 4: diabetic rats treated with 10 mg kg^−1^ Synacinn™.

Group 5: diabetic rats treated with 50 mg kg^−1^ Synacinn™.

Group 6: diabetic rats treated with 100 mg kg^−1^ Synacinn™.

Group 7: diabetic rats treated with 250 mg kg^−1^ Synacinn™.

Group 8: diabetic rats treated with 250 mg kg^−1^ twice daily (b.i.d.) Synacinn™.

Group 9: diabetic rats treated with 6 mg kg^−1^ glibenclamide.

Freshly prepared extracts and glibenclamide were orally administrated once daily, except for group 8, which received treatment twice daily, for 28 days. The administration volume was 1 mL, and the animals were carefully monitored every day and weighed every week. Food consumption and water intake data were recorded daily.

#### 4.3.3 Assessment of serum parameters

Prior to blood collection, rats fasted overnight and were anesthetized using a ketamil–xylazil cocktail. Blood was drawn through the tail vein and allowed to clot. Serum was separated *via* centrifugation at 4000 rpm for 10 minutes at 4 °C. Fasting blood glucose levels were determined using a glucometer (Gluco Dr Auto, All Medicus, Korea). Meanwhile, TG, TC, ALT, ALP, creatinine, and uric acid levels were analyzed using an Architect Ci8200 machine (Abbot, US).

#### 4.3.4 Histological analysis

Upon dissection, livers, kidneys, and pancreases were removed from the carcasses. Samples were fixed with 10% buffered formalin for 24 hours before the dehydration process using a tissue processing machine. Then, samples were embedded into molds with paraffin wax and sectioned at 5 μm. The islet areas were measured *via* selecting the six largest pancreatic islets/group in all groups using a Leica™ DM LB2 light microscope (Germany) that was equipped with the Leica™ Image Analyzer System. The LAS 4.0 system software and a ×20 objective lens were used to measure the area (μm^2^) of the islets of Langerhans.

### 4.4 Phytochemical quantification *via* HLPC

HPLC analysis was conducted using a Waters 2690 Alliance Separation Module with a LiChrospher® 100 RP-18 endcapped Merck column cartridge (250 × 4.6 mm, 5 μm). For the gradient flows used for gallic acid and catechin, the two-solvent system (solvent A: 0.1% formic acid in water; solvent B: 0.06% formic acid in water) was as follows: 0 min: 95% A, 5% B; 10 min: 80% A, 20% B; 22 min: 80% A, 20% B, which was held until the end. For andrographolide, methanol : water (60 : 40) was used; for rosmarinic acid, 0.5% phosphoric acid in water and acetonitrile (60 : 40) were used; and for curcumin, 5% acetic acid in water and acetonitrile (50 : 50) were used. The flow rate for all mobile phases was 1.0 mL min^−1^. The chromatograms were monitored at wavelengths of 340 nm (rosmarinic acid), 272 nm (gallic acid), 223 nm (andrographolide), 280 nm (catechin), and 428 nm (curcumin).

### 4.5 *In silico* analysis with the glucokinase enzyme

The crystal structure of human glucokinase (PDB ID: 1V4S) was obtained from the Protein Data Bank (PDB) in the form of a 3D structure as a target receptor for docking studies. The energy of this protein was minimized using the ModRefiner program.^72^ The 5YE8 protein residues were removed and separated. The grid dimensions were set at 25.5 × 1.8 × 68.0, according to the coordinates *x*, *y*, and *z*, for the target binding sites. Hydrogen bond computing Gasteiger charges were added to the protein using autodock tools. The ligand structures of andrographolide, gallic acid, curcumin, catechin, and rosmarinic acid were obtained from the PubMed Database (https://www.ncbi.nlm.nih.gov/pubmed/). Molecular docking simulations were performed using Autodock Vina.^73^ AutoDock Vina was used for finding the ligand conformation with the lowest predicted free energy of binding. The results were analyzed using Discovery Studio Visualizer and Chimera to view and represent the molecular interactions.

## 5. Conclusions

The current findings conclude that Synacinn™ exerts anti-hyperglycemic activity *via* effectively lowering the blood glucose, total triglyceride, and cholesterol levels in STZ-induced type 1 diabetes rats. Synacinn™ at 250 (b.i.d.) mg kg^−1^ improves the pathophysiological symptoms of diabetes, such as polyphagia, polydipsia, and weight loss. It also significantly decreases serum ALT, ALP, and creatinine levels after 28 days of consumption. Histopathological analysis revealed the protective effects of Synacinn™ on the liver, kidneys, and pancreas. Therefore, based on the above characteristics and the superior effects compared to the other tested doses, 250 (b.i.d.) mg kg^−1^ Synacinn™ is suggested to be an appropriate dose to act as an alternative drug for the management of diabetes mellitus.

## Conflicts of interest

The authors declare that they have no conflicts of interest.

## Supplementary Material
